# Supplementary material for: The Effects of Foam Rolling Training on Performance Parameters: A Systematic Review and Meta-Analysis including Controlled and Randomized Controlled Trials
Source: Int J Environ Res Public Health. 2022 Sep 15;19(18):11638. doi: 10.3390/ijerph191811638 (PMC9517147; doi:10.3390/ijerph191811638)
Supplement: Supplementary file 1 [file ijerph-19-11638-s001.zip › ijerph-1881856-supplementary.pdf]

Table S1. Search codes in the respective databases.

Database	Search code
Scopus	TITLE-ABS-KEY (("Chronic effects" OR "training effects" OR effects OR "long-term" OR intervention) AND ("foam rolling" OR "self-myofascial release" OR "roller massage" OR "foam roller") AND ("performance" OR "strength" OR "force" OR "hypertrophy" OR "power" OR "torque" OR "height" OR "RFD" OR "rate of force development" OR "jump" OR maximum OR maximal))
Pubmed	("Chronic effects"[Title/Abstract] OR "training effects"[Title/Abstract] OR effects[Title/Abstract] OR "long-term"[Title/Abstract] OR intervention[Title/Abstract]) AND ("foam rolling"[Title/Abstract] OR "self-myofascial release"[Title/Abstract] OR "roller massage"[Title/Abstract] OR "foam roller"[Title/Abstract]) AND ("performance"[Title/Abstract] OR "strength"[Title/Abstract] OR "force"[Title/Abstract] OR "hypertrophy"[Title/Abstract] OR "power"[Title/Abstract] OR "torque"[Title/Abstract] OR "height"[Title/Abstract] OR "RFD"[Title/Abstract] OR "rate of force development"[Title/Abstract] OR "jump"[Title/Abstract] OR maximum[Title/Abstract] OR maximal [Title/Abstract]))))
Web of Science	TS=(("Chronic effects" OR "training effects" OR effects OR "long-term" OR intervention) AND ("foam rolling" OR "self-myofascial release" OR "roller massage" OR "foam roller") AND (“performance” OR “strength” OR “force” OR “hypertrophy” OR “power” OR “torque” OR “height” OR “RFD” OR “rate of force development” OR “jump” OR maximum OR maximal))
Cochrane Library	(("Chronic effects" OR "training effects" OR effects OR "long-term" OR intervention) AND ("foam rolling" OR "self-myofascial release" OR "roller massage" OR "foam roller") AND (“performance” OR “strength” OR “force” OR “hypertrophy” OR “power” OR “torque” OR “height” OR “RFD” OR “rate of force development” OR “jump” OR maximum OR maximal))
